# [^18^F]-fluoro-D-deoxyglucose positron emission tomography findings in Kaposi sarcoma herpes virus associated multicentric Castleman disease: correlation with clinical, inflammatory, and virologic parameters

**DOI:** 10.1186/1750-9378-7-S1-P48

**Published:** 2012-04-19

**Authors:** Mark N Polizzotto, Corina Millo, Thomas S Uldrick, Karen Aleman, Millie Whatley, Kathleen M Wyvill, Deidre O’Mahony, Vickie Marshall, Denise Whitby, Seth M Steinberg, Richard F Little, Robert Yarchoan

**Affiliations:** 1 HIV and AIDS Malignancy Branch, Center for Cancer Research, National Cancer Institute, National Institutes of Health, Bethesda, MD, USA; 2 PET Department, Clinical Center, National Institutes of Health, Bethesda, MD, USA; 3 Viral Oncology Section, AIDS and Cancer Virus Program, SAIC-Frederick, National Cancer Institute, National Institutes of Health, MD, USA; 4 Biostatistics and Data Management Section, Center for Cancer Research, National Cancer Institute, National Institutes of Health, Bethesda, MD, USA

## Background

KSHV-associated multicentric Castleman disease (KSHV-MCD) is a lymphoproliferative disorder associated with severe inflammatory symptoms, cytopenias and biochemical abnormalities. Improved techniques to assist diagnosis and aid monitoring are required. We prospectively assessed ^18^FDG-PET/CT findings in KSHV-MCD in relation to clinical symptoms and markers of disease activity.

## Methods

Patients enrolled on a natural history study of KSHV-MCD underwent ^18^FDG-PET/CT at disease activity, except where unstable, and at complete clinical and biochemical remission. ^18^FDG-PET/CT was evaluated blind to clinical status. Symptoms, C-reactive protein (CRP), HIV viral load (VL) in plasma and KSHV VL in peripheral blood mononuclear cells were assessed. Associations with ^18^FDG-PET/CT maximal standardized uptake value (SUV_max_) were explored using Spearman correlations (CRP, symptoms, log_10_[KSHV VL]) or exact Wilcoxon rank sum (HIV VL, detectable or not).

## Results

26 patients (24 male, median age 43 [range 34-56], all with HIV) were studied. In 3, we identified intercurrent lymphoma; these were excluded from the primary analysis. The remaining 23 underwent 19 studies during disease activity (16 symptomatic, 3 with laboratory manifestations only), and 21 studies at remission.

In symptomatic patients, ^18^FDG-PET showed symmetrical hypermetabolic adenopathy (diffuse in 15 [94%], focal in 1 [6%]) and increased splenic metabolic activity with splenomegaly (abnormal in 14 [93%] of the 15 with intact spleens). Marrow and hepatic abnormalities were less common and mild. In patients with laboratory manifestations only, 2 (66%) had mild splenomegaly and limited adenopathy and 1 (33%) isolated adenopathy.

During disease activity, median SUV_max_ was 6 (2-8), and was associated with symptom severity (R=0.61 p=0.005), CRP (R=0.54, p=0.017) and KSHV VL (R=0.56, p=0.013), but not HIV VL (p=0.69). Intercurrent lymphomas (2 PEL and 1 diffuse large B-cell) demonstrated intensely hypermetabolic abnormalities involving restricted asymmetrical sites, with median SUVmax 11 (range 7–38). At remission, 11 (53%) had normal ^18^FDG-PET/CT; 10 (47%) had minor nodal abnormalities and 4 (19%) mildly increased splenic metabolism without splenomegaly. Intercurrent pathologies contributed to some abnormalities. One had progressive increase in splenic and nodal SUV_max_ over 3 scans (not included in primary analysis) before relapse.

## Conclusion

^18^FDG-PET/CT demonstrated widespread nodal and splenic abnormalities during disease activity, improving with remission. Subclinical disease may also be detectable. Findings were distinguishable from suppressed HIV or intercurrent lymphoma by intermediate metabolic intensity and diffuse anatomic distribution. SUV_max_ was associated with symptom severity, systemic inflammation, and KSHV burden. ^18^FDG-PET/CT may be a useful non-invasive adjunct in the diagnosis and monitoring of KSHV-MCD.

